# Protease-activated receptors (PARs)—biology and role in cancer invasion and metastasis

**DOI:** 10.1007/s10555-015-9599-4

**Published:** 2015-11-16

**Authors:** Marek Z. Wojtukiewicz, Dominika Hempel, Ewa Sierko, Stephanie C. Tucker, Kenneth V. Honn

**Affiliations:** Department of Oncology, Medical University of Bialystok, 12 Ogrodowa St., 15-025 Bialystok, Poland; Department of Clinical Oncology, Comprehensive Cancer Center , Bialystok, Poland; Department of Radiotherapy, Comprehensive Cancer Center, Bialystok, Poland; Bioactive Lipids Research Program, Department of Pathology-School of Medicine, Detroit, MI USA; Department of Chemistry, Wayne State University, Detroit, MI USA; Department of Oncology, Karmanos Cancer Institute, Detroit, MI USA

**Keywords:** PAR-1, PAR-2, Tissue factor, Thrombin, Cancer invasion, Metastasis, Microenvironment

## Abstract

Although many studies have demonstrated that components of the hemostatic system may be involved in signaling leading to cancer progression, the potential mechanisms by which they contribute to cancer dissemination are not yet precisely understood. Among known coagulant factors, tissue factor (TF) and thrombin play a pivotal role in cancer invasion. They may be generated in the tumor microenvironment independently of blood coagulation and can induce cell signaling through activation of protease-activated receptors (PARs). PARs are transmembrane G-protein-coupled receptors (GPCRs) that are activated by a unique proteolytic mechanism. They play important roles in vascular physiology, neural tube closure, hemostasis, and inflammation. All of these agents (TF, thrombin, PARs—mainly PAR-1 and PAR-2) are thought to promote cancer invasion and metastasis at least in part by facilitating tumor cell migration, angiogenesis, and interactions with host vascular cells, including platelets, fibroblasts, and endothelial cells lining blood vessels. Here, we discuss the role of PARs and their activators in cancer progression, focusing on TF- and thrombin-mediated actions. Therapeutic options tailored specifically to inhibit PAR-induced signaling in cancer patients are presented as well.

## Introduction

The association between blood coagulation, with respect to venous thrombosis and cancer development was first described in the nineteenth century by Drs. Trousseau and Bouillaud [1]. Trousseau’s syndrome is a prominent example of cancer-related thromboembolism. The additional proof that coagulation factors actively participate in cancer invasion is the fact that anticoagulants improve cancer patients’ survival [2–4]. The dissemination of cancer from the primary tumor is the main cause of cancer-related death and an enormous clinical challenge [5]. Therefore, the contribution of the coagulation factors to the mechanisms of metastasis has been the subject of intensive study. According to existing data, reciprocal interactions between cancer cells, extracellular matrix proteins, as well as endothelial cells (ECs) and blood cells, play a pivotal role in tumor invasiveness and metastasis [4–6]. The complex system of enzymes, bioactive lipids, cytokines, and receptors is indispensable for efficient intracellular communication on local and distant ground. It appears that coagulants, serine proteases, and matrix metalloproteases (MMPs) facilitate tumor cell metastasis by modulating a number of host vascular cell responses as well as by acting directly on tumor cells themselves. Since the 1960–1970s, hormone-like effects of proteases in target tissues have been recognized, e.g., insulin-like effects of pepsin or chymotrypsin as well as mitogenic actions of thrombin and trypsin at the cell membrane [7, 8]. Tissue factor (TF) and thrombin are present in both the tumor microenvironment and malignant cells [9–11]. Tissue factor in a complex with coagulation factor VIIa (FVIIa) initiates blood coagulation leading to thrombin generation. In addition to cleaving fibrinogen, thrombin activates cells through a unique proteolytic mechanism [12]. Thrombin opens its receptor active site by cleavage of a key extracellular domain (residues LDPR/S). This hinge-like mechanism led to the receptor being referred to as the tethered ligand thrombin receptor (TLTR) [13]. Collectively, the thrombin-activated receptors have come to be referred to as protease-activated receptors (PARs). In the late twentieth century, pioneering work identified the presence of the G-protein-coupled thrombin receptor at the surface of cancer cells in solid tumors [14]. The predominant activators of PARs in cancer cells are thrombin, MMPs, trypsin, TF, FVIIa, FXa, and their ternary complex TF/FVIIa/FXa [5, 15, 16]. The expression of PARs (mainly PAR-1 and PAR-2) is implicated in the development of several types of human malignant cancers and correlates directly with the degree of invasiveness exhibited by both primary and metastatic tumors [17, 18]. As a growing number of studies have documented the role of PARs in cancer cell proliferation and tumor growth, considerable effort has been devoted to development of both protease inhibitors (functional) and receptor active site inhibitors (pharmacological PAR antagonists) [16, 19–22].

Here, we provide a comprehensive review of the contribution of PARs to cancer invasiveness and dissemination by focusing on actions elicited by TF and thrombin in tumors, ECs, and blood cells. We also present potential therapeutic strategies targeted to interactions induced via PAR-associated signaling.

## Protease-activated receptors

### Discovery, localization, and activators

PARs are transmembrane G-protein-coupled receptors (GPCRs) [23, 24]. Each of four PARs—PAR-1, PAR-2, PAR-3, and PAR-4—are encoded by distinct genes. PAR-1, the first receptor to be discovered, was identified in 1991 by two independent laboratories in search of the GPCR that mediated thrombin signaling in human and hamster cells. Microinjection into Xenopus oocytes of RNA transcribed *in vitro* from the cDNA of a novel putative receptor led to the expression of a functional thrombin receptor [25, 26].

PAR-2, which is activated by trypsin, was identified by screening a mouse genomic library for GPCRs with oligos based on conserved transmembrane regions of the bovine substance K receptor [27]. Subsequently, PAR-3 and PAR-4 were cloned by mRNA screening of rat platelets and by investigating a human expressed sequence tag database, respectively [28, reviewed in 29].

PARs are expressed on nearly all cell types in the blood vessel wall (ECs, fibroblasts, myocytes) and blood (platelets, neutrophils, macrophages, leukemic white cells) with exception of red blood cells [15]. Thrombin-activated PAR-1, PAR-3, and PAR-4 are also expressed in epithelium, neurons, astrocytes, and immune cells [15, 23, 29–31]. PAR-2, which is activated by trypsin-like serine proteases, is found in human vascular, intestinal, neuronal, and airway cells. Its expression increases in injured tissues or after stimulation by inflammatory mediators [29, 30, 32]. Most cells express multiple PARs that are fully functioning with respect to signal capacity. However, many form heterodimers where they reciprocally function as cofactors to potentiate protease activity, thereby leading to transactivation of one receptor by another to give a cellular response [30, 33]. For example, thrombin binds and cleaves PAR-3 in murine platelets without eliciting further cellular signaling from PAR3, but this facilitates activation of the low-affinity thrombin receptor, PAR-4 [30, 34]. This exceptional mechanism of transactivation exists between PAR-1 and PAR-2, or PAR-1 and PAR-4 in human endothelial cells or platelets, respectively. Tethered ligand of one receptor, generated by thrombin-mediated proteolysis, can directly stimulate the active site of another PAR and effectively induce intracellular signaling [33, 35, 36]. It seems that PARs form physical heterodimers, especially after stimulation by cytokines during inflammation [33]. Response activation by heterodimers, e.g., PAR-1/PAR-2 is distinct from that elicited by homodimers, as evidenced by early, barrier-disruptive (PAR-1 dominant), and late, barrier-protective (PAR-1/PAR-2 dominant) stages of sepsis [33]. There are many other activators of PARs in addition to thrombin and trypsin (Table 1).

**Table 1:** Proteases leading to protease-activated receptor (PAR) activation

	PAR-1	PAR-2	PAR-3	PAR-4
Proteases	Thrombin Factor Xa TF-VIIa-Xa APC Plasmin Granzyme A Gingipains-R Trypsin MMP-1, MMP-9, MMP-2, MMP-13, MMP-14	Trypsin Tryptase Factor VIIa Factor Xa TF-VIIa-Xa TF-VIIa MT-SP1 Proteinases-3 Gingipains-R Kallikrein 14	Thrombin APC	Thrombin Plasmin Cathepsin G Trypsin Factor Xa Gingipains-R Kallikrein 14

Source: [15, 29, 32, 37–40]

*APC* activated protein C, *MT-SP1* membrane-type serine protease 1, *MMPs* matrix metalloproteinases

Soon after the discovery of the thrombin receptor on normal human tissues, biologically functional receptor was also demonstrated in human cancer cells [14, 41, 42]. Additional studies then discovered PAR-1 (Table 2) and PAR-2 (Table 3) expression on several cancer cell lines, including epithelial carcinomas, melanoma, glioblastoma (GBM), and sarcoma [16, 31, 37, 43–73]. Importantly, PAR-1 expression was also described in cancer-associated fibroblasts (in contrast to benign lesions, where such expression was not observed), ECs, myocytes of vessels, mast cells, and macrophages in the malignant tumor microenvironment [32, 74], where PAR-1 and PAR-2 stimulate macrophages to synthesize and secrete thrombin as well as other growth factors [74].

**Table 2:** Protease-activated receptor 1 (PAR-1) expression and activation in cancer settings

Cancer cell line/xenograft	Activator	Cellular effect
Nasopharyngeal CNE1-LMP1 [43]	Thrombin SFLLRN	Invasion, tumor growth
Gastric MKN45/PAR1 MKN74 cells [44]	Thrombin	EMT Increased Snail, fibronectin expression Decreased E-cadherin expression
Gastric MKN45/PAR1 [45, 46]	Thrombin	NF-κB activation Increased EGFR, cytoskeletal protein expression Increased cell proliferation, motility Matrigel barrier invasion
Breast xenograft [47]	Thrombin	EGFR and ErbB signaling, transactivation Invasion Tumor growth
Melanoma murine model B16F10 [48]	Thrombin	Pulmonary metastasis
Chondrosarcoma [49]	Thrombin	Increased expression of MMPs Cell migration
Melanoma [50]	Thrombin	Cell motility, migration
Medulloblastoma [51]	Thrombin	Increased IL1β, chondromodulin 1 (LECT1) expression
Glioblastoma [31] U178MG	TFLLR-NH2	Increased Ca^2+^ levels
Breast MDA-MB-231 [16]	MMP1	Activation of Akt survival pathway
Peritoneal ovarian cancer xenograft [39]	MMPs	Angiogenesis, metastases formation
Breast xenograft [52]	MMP1	Invasion, migration

*MMP* matrix metalloproteinase, *EMT* epithelial–mesenchymal transition, *SFLLRN* PAR-1 activating peptide, *EGFR* epidermal growth factor receptor

**Table 3:** Protease-activated receptor 2 (PAR-2) expression and activation in cancer settings

Cancer cell line/xenograft	Activator	Cellular effect
Pancreas SW 1990 [53]	Trypsin SLIGKV	Proliferation, invasion, migration Increased mRNA expression of MMP-2
Pancreas SW1990, Capan-2, Panc-1 Xenografts [54]	Trypsin SLIGKV	Increased MAPK activity Proliferation Tumor growth
Pancreas MIA PaCa-2 [55]	Trypsin SLIGRL	Ca^2+^ immobilization, increase in inositol (1,4,5) triphosphate level, protein kinase activation, decrease in DNA synthesis
Esophageal EC109 [56]	Trypsin	Invasion, metastasis
Hepatoma HepG2 cells [57]	Trypsin SLIGKV	Proliferation
Hepatocellular [58]	Trypsin	Invasion through Matrigel barrier
Cholangiocarcinoma [59]	Trypsin	Invasion through collagen membrane barrier
Colorectal [60]	Trypsin	Invasion, metastasis
Colon DLD-1 [61, 62]	Trypsin SLIGKV	Proliferation
Cervical UISO-SQC-1, HeLa, SiHa, CasKi and C-33 A [63]	Trypsin	Proliferation
Oral squamous ZK-1 [64]	SLIGRL	Proliferation
Meningioma [65]	Trypsin SLIGRL	Ca^2+^ immobilization,
Breast MDA-MB-436, ZR-75-1 [66]	Trypsin SLIGRL	Migration in a chemokinesis mechanism
Breast Adr-MCF-7 [67]	TF/VIIa/Xa	Migration
Breast MDA-MB-231 [68]	TF/FVIIa/ FXa	Migration, invasion
Breast MDA-MB-231 [38]	FVIIa	Transcription of 39 genes: cytokines, chemokines, growth factors involved in tumor development, metastasis and angiogenesis
Breast MDA-MB-231 [69]	TF/FVIIa	Increased IL-8 expression
Breast MCF-7 [70]	TF	Increased MMP-9 expression
Lung A549 cells [71]	MMP-1	Increased MCP-1 expression
Glioma cell U87-MG and HOG [72]	AP	Increased IL-8, VEGF expression
GBM cell lines A172 and U87-MG [73]	AP	Increased VEGF expression

*AP* agonist peptide, *MMP* matrix metalloproteinase, *MAPK* mitogen-activated protein kinase, *SLIGKV*, *SLIGRL* PAR-2 activating peptides, *TF* tissue factor, *aFVII* active factor VII, *aFX* active factor X

*In vitro* experiments revealed that overexpression of PARs in cancer cells was the result of increased transcriptional activity, and not gene amplification [75]. *PAR-1* expression in epithelial tumors is elevated by the transcription factor Egr-1, but inhibited by the tumor suppressor *p53* [75]. In melanoma, the *PAR-1* gene is differentially regulated by activator protein-2α that binds to the *PAR-1* promoter in low- and nonmetastatic melanoma cell lines, and SP-1 transcription factors that are active in metastatic melanoma cell lines [76, 77]. There are also known polymorphisms of the *PAR-1* gene that are associated with worse prognosis in lung cancer (*PAR-1* -14 Ivs A/A), in pancreatic cancer (*PAR-1* -506 Ins/Del) and in gastric cancer (*PAR-1*-505 Ins/Del) [17, 78, 79].

### Mechanism of activation

#### Canonical (standard) activation of PARs

**Fig. 1:**
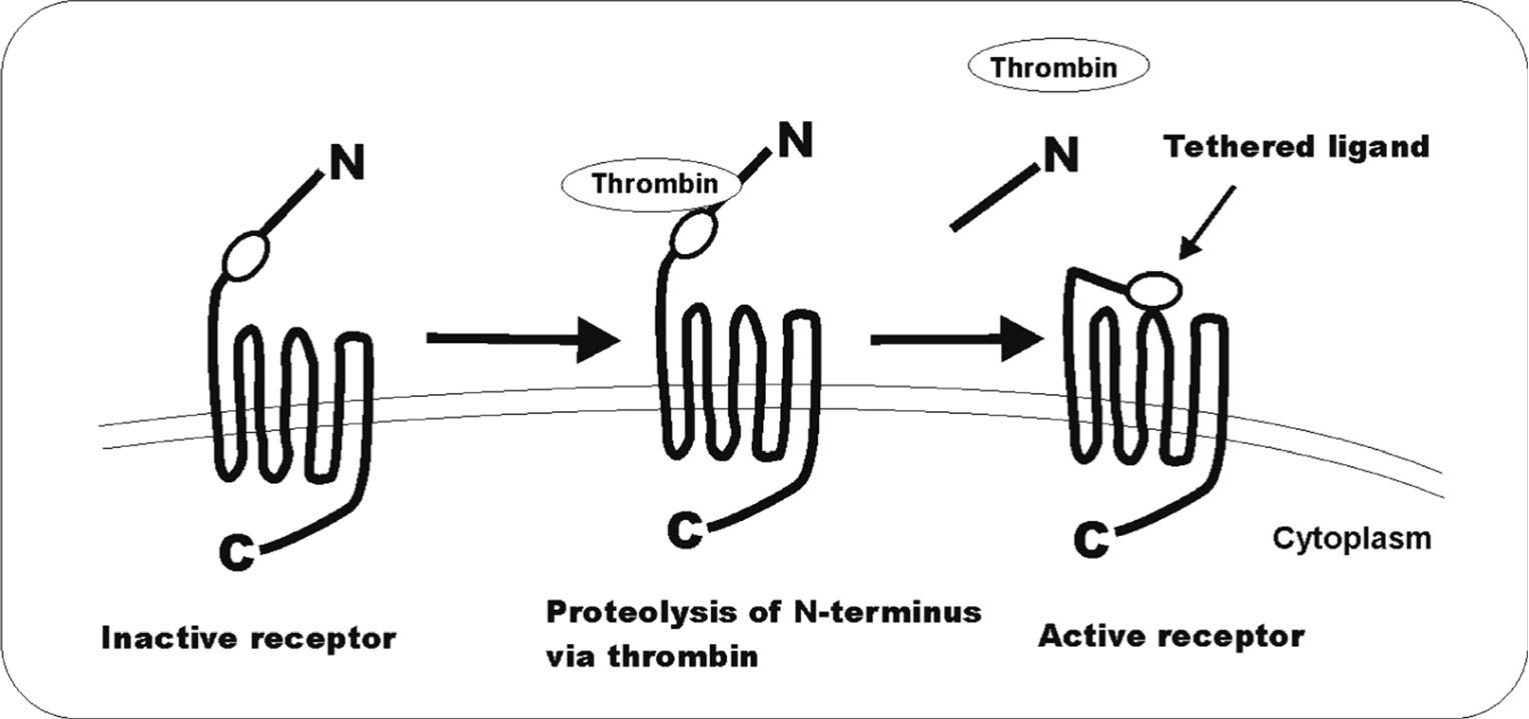
Structure and mechanism of protease-activated receptor (PAR) activation

PARs are composed of seven transmembrane α-helices, a cytoplasmic domain for G-protein coupling, and an extracellular N-terminus sequence (Fig. 1). The mechanism of PAR activation was most thoroughly investigated for PAR-1 [30, 80]. The predominant activator of PAR-1, thrombin, binds to the receptor N-terminus LDPR^41−^S^42^ sequence and cleaves the R^41−^S^42^ peptide bond [12]. The new, unmasked sequence generated this way acts as a tethered ligand that binds in an intramolecular fashion to residues ^42^SFLLRN^47^ in the conserved region of the second loop of the receptor to induce transmembrane signaling (Fig. 1) [29]. The sequence of the tethered ligand is distinct and characteristic for each of the PARs.

The affinity of thrombin for PAR-1 is sufficient to induce transmembrane signaling in the absence of any cofactors. However, for activated protein C (APC) protease to cleave the N-terminus of PAR-1 in cancer cells, the endothelial protein C receptor (EPCR) is required as a cofactor [81]. Therefore, the need for cofactors is dependent on context and which protease is acting on the PAR in question.

Cleavage of PAR-1 and PAR-3 is facilitated by interactions between the thrombin exosite I and a hirudin-like acidic element located in the C-terminus of the tethered PAR sequence. Mutational analysis of the thrombin cleavage site identified the P4-L^38^ and P2-P^40^ residues as critical for proper cleavage of PAR-1 [15]. These highly acidic regions increase thrombin affinity so that lower concentration of protease is needed for PAR-1 activation compared to PAR-4, which does not have such a domain and so requires either higher levels of thrombin, or support of its co-receptors, PAR-1 or PAR-3, which in turn then facilitate PAR-4 association with thrombin at low concentration [30, 36]. In addition, PAR-4 also contains an anionic cluster (D^57^D^59^E^62^D^65^) in its exodomain that slows the dissociation rate of PAR-4 from the cationic thrombin [15]. Proteolysis of the R^47^-G^48^ peptide bond of PAR-4 by thrombin then generates the appropriate ligand [82].

PAR-2 is activated by trypsin-like serine proteases or coagulation factors (Table 1) [83]. Proteolysis of the R^34−^S^35^ peptide bond in the PAR-2 N-terminus induces further signaling [27]. In contrast to PAR-1 and PAR-3, PAR-2 lacks the exosite recognition sequence [84]. Instead, glycosylation of residues located in the N-terminus (for FVIIa: Q^40^, D^72^, Q^143^, and T^151^–S2’ subsite) as well as other cofactors serve as key regulators of interactions between proteases and PAR-2. FVIIa recognizes PAR-2 predominantly by catalytic cleft interactions, while the S2’ pocket accommodates the side chain of PAR-2 L^38^, P2’ [84]. Activation of PAR-2 by the TF/FVIIa binary complex involves cellular pools of TF with low affinity for FVIIa, whereas high affinity cell surface TF mediates coagulation activation and the associated cell signaling of the ternary complex of TF/FVIIa/FXa [84]. In the latter complex, FXa is the primary activator of PAR-2. In some breast cancer cell lines, FXa may solely activate PAR-2 [68].

**Fig. 2:**
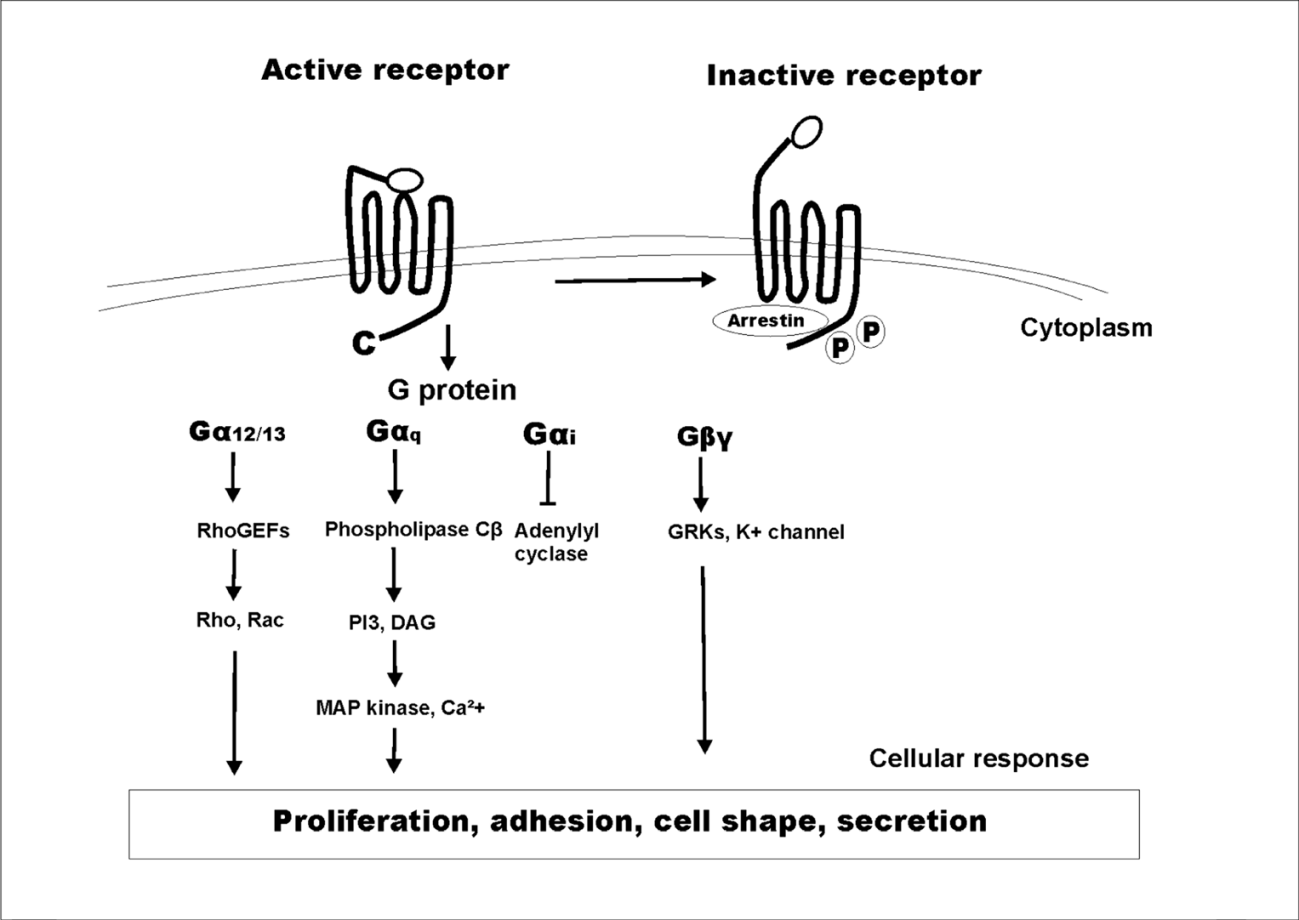
Protease-activated receptor 1 (PAR-1) activation and signaling. Protein RhoGEFs, Rho, Rac. *MAP kinase* mitogen-activated protein kinase, *DAG* diacylglycerol, *PI3* inositol (1,4,5)-trisphosphate, *GRKs* G-protein-coupled receptor kinases

PAR-induced cellular signaling may be activated through G-protein interaction or arrestin association and depends on the type of receptor. PAR-1 conformational changes mediated by thrombin result in receptor coupling to Gα protein (Gαq, Gαi, and Gα_12/13_) and Gβγ. Heterotrimers composed of PAR-1 and Gαq lead to activation of mitogen-activated protein (MAP) kinase and increased Ca^2+^concentration, while complexes of PAR1 with Gα_12/13_ activate the small G-proteins, Rho and Rac [23, 85]. PAR activation also induces signaling cascades associated with protein kinase C and tyrosine kinases. Heterotrimers with Gβγ promote phosphatidylinositol 3-kinase (PI3-K) activation while complex with Gαi inhibits adenylyl cyclase (AC) (Fig. 2). PAR-4 and PAR-2 also interact with G-proteins, in contrast to PAR-3, which does not [75]. PAR-2 may also interact with β-arrestin (a multifunctional adaptor protein), which binds complex TF/FVIIa to mediate PAR-2 activation [40]. The intracellular second messengers that are activated by this interaction are ERK-1 and ERK-2 (extracellular signal-regulated kinase-1/2) [33, 86].

Finally, the activation of PARs increases transcription of cytokines, chemokines, and growth factors and production of bioactive lipids to regulate cell proliferation, apoptosis, adhesion, and migration, which results in tumor growth, invasion, and metastasis (Fig. 2). Activation of distinct subtypes of PARs is cancer-specific, for example, gene regulation elicited by TF/FVIIa through PAR-2 in MDA-MB-231 cells may occur in glioblastoma cell lines through thrombin-mediated activation of PAR-1 [38]. These findings show how specific protein-protein interactions contribute to PAR activity and explain the diversity of cellular responses in cancer.

#### Noncanonical activation of PARs

Cleavage of the extracellular portion of the PAR-1 receptor by thrombin occurs at a canonical R^41^-S^42^ site, while MMP-1 cleaves PAR-1 at a novel site (D^39^-P^40^) resulting in a tethered ligand that is two amino acids longer (PR-SFLLRN) than that generated by thrombin. The noncanonical tethered ligand preferentially activates G_12/13_, Rho-GTP, and MAPK pathways, leading to changes in shape and motility of platelets [15, 87]. MMP-specific signaling patterns exhibited by PAR-1 are known as biased agonism and produce distinct functional output by the cell. Furthermore, studies with breast cancer cell lines have shown that activation of secondary signaling in the canonical and noncanonical models is operational at different times. Peak Akt signaling occurs after 5 min in thrombin-triggered activation, while MMP-1-triggered Akt activation occurs after 1 h [16]. Similar kinetic differences for MAP kinase activation were observed during differential cleavage by these two enzymes [15]. Recently, another model of PAR functional selectivity based on canonical versus noncanonical activation has been discovered [88]. In this study, it was determined that noncanonical PAR3 activation by FXa led to tunica intima endothelial receptor tyrosine kinase 2 (Tie2) activation that was also dependent on endothelial protein C receptor (EPCR), and ultimately vascular protection by upregulation of zona occludens 1 (ZO-1) to stabilize cell-cell junctions. This protective effect by noncanonical activation is in direct contrast to canonical activation of PAR3 by thrombin, which leads to vascular barrier permeability.

### Regulation of PARs activity

The activation of PARs is irreversible. Therefore, precise regulation of PARs is of paramount importance. Inactive (uncleaved pro-form) PAR circulates constitutively between the plasma membrane and intracellular compartment providing a steady pool of PAR at the cell surface. PAR-1 internalization requires previous ubiquitination as well as binding to the clathrin/AP2 (adapter protein 2) complex and dynamin [89]. Trafficking of PAR-1 to lysosomes occurs after recognition by the sorting protein sorting nexin-1 (SNX1) [90].

Two major mechanisms regulate activated (cleaved) PAR, namely desensitization or receptor trafficking [32]. Phosphorylation of PAR-1 through G-protein-coupled receptor kinases (GPCRKs, GRKs) and arrestin binding ensures G-protein uncoupling and PAR-1 desensitization within seconds (rapid mechanism). However, arrestin is unnecessary for internalization. PAR-1 is then internalized/mobilized from the cell membrane to the endosomes and subsequently degraded in lysosomes [91, 92]. Slower degradation of PAR-4 compared to PAR-1 and lack of its rapid phosphorylation lead to longer PAR-4 activity, which is pivotal for thrombin-mediated platelet aggregation [91]. PAR-3 deactivation is also phosphorylation-dependent. The regulation of PAR-2 activity also occurs by arrestin-mediated uncoupling followed by receptor internalization. The extent to which phosphorylation plays a role in this case in unclear. An additional mechanism has been reported whereby MMP-1 may desensitize PARs by cleaving the N-terminal exodomain with part of the tethered ligand sequence [93].

In contrast to regulation of PARs in normal tissues, there is constitutive PAR activation in cancer cells resulting in sustained stimulation of second messenger signaling (e.g., Erk1/2-dependent) [94]. In metastatic breast carcinoma cells, it was demonstrated that proteolytically activated PAR-1 was not sorted to lysosomes and degraded, which resulted in persistent signaling that promoted breast cancer invasion [94].

PARs activity is indirectly regulated by inhibition of their activators. In this manner tissue factor pathway inhibitor (TFPI) disables PARs cleavage by forming a complex with TF/FVIIa/FXa that leads to its internalization and blockade of thrombin generation [95, 96]. Tissue factor pathway inhibitor-2 (TFPI-2) not only blocks TF/VIIa complex but also plasmin and trypsin (PARs activators) and thereby indirectly affects the conversion of pro-MMP-1 into its active form (also PARs activator) [95]. Collectively, these data suggest that TFPI may impede PARs activity and may provide therapeutic value.

## PARs and proteases—cooperation in cancer progression

### Tissue factor

TF is a membrane glycoprotein present on subendothelial cells that initiates blood coagulation. The disruption of endothelium exposes TF to coagulation factors present in the bloodstream. TF binds to FVII and causes its activation (FVIIa). The TF/FVIIa complex may further activate FX (FXa), which together with its cofactor FVa, generates thrombin (FIIa) from prothrombin by proteolytic conversion. Thrombin initiates coagulation by platelet activation and fibrin conversion from fibrinogen, resulting in effective blood clotting [83].

TF is also the most prominent procoagulant of cancer cells and is a determinant of tumor progression [97]. TF has been discovered on the surface of distinct malignant cells, tumor vasculature, and tumor microenvironment: stem cells, macrophages, ECs, and myofibroblasts [9, 10, 40, reviewed in 98, 99]. It is also widely recognized that TF expression correlates with greater invasiveness and higher clinical stage of the malignant disease and is associated with poor overall prognosis [reviewed in 40, 97]. Tumor cells endogenously express TF constitutively, or they induce production of TF in their surroundings by producing soluble substances capable of triggering monocytes and ECs to express it [98]. TF expression is associated with carcinogenic events during oncogenic transformation, as there exists mounting evidence that mutations of proto-oncogenes and tumor suppressor genes influence its expression [40]. In colorectal cancer, the K*-ras* and *p53* mutations eliciting the MAPK and PI3-mediated signaling pathways result in enhanced expression of TF [100]. In lung cancer, similar observations were made for PTEN and *p53* mutations [101]. TF expression has also been shown to be modulated in other cancers by constitutively active mutant forms of epidermal growth factor receptor (EGFRvIII) in glioma and vulva cells, as well as Src family kinases, TGF-β production, and hypoxia [40].

There are numerous mechanisms by which TF impacts cancer biology. First, upon activation by factors VII and X and creating complexes with them (TF/VIIa, TF/Xa, TF/VIIa/Xa), TF promotes PAR-1- and PAR-2-mediated signaling responsible for the proliferative response of cancer cells [38, 97, 102]. In addition, TF may directly signal via its cytoplasmic tail through Rac1 and p38 and cytoskeletal remodeling [103]. Furthermore, an alternatively spliced isoform of TF (asTF) also affects tumor growth independently of VIIa and PARs cleavage, through the activation of integrins α_6_β_1_ and α_V_β_3_ on ECs to promote angiogenesis [97, 104]. Human asTF promoted tumor growth and angiogenesis in pancreatic cancer [105] but was inactive in the coagulant-dependent mechanism of metastasis in a breast cancer model [106].

In experimental and clinical models, cancer cells expressing TF had greater tendency to metastasize compared to TF-deficient cells [106]. TF effects on metastasis may be mediated via mechanisms that are either dependent on or independent of coagulation activation, i.e., through TF signaling function. Tissue factor likely promotes proliferative and infiltrative potential rather than adhesive properties of metastatic cells [30, 68, 107]. There is also evidence that TF plays a role in tumor cell intravasation, which is the first step in dissemination of malignant cells [108].

Although TF may promote both PAR-1 and PAR-2 activation, it seems that TF or TF/FVIIa complex typically triggers PAR-2 but not PAR-1 signaling in cancer cells [38, 67–69, 99, 102, 109, 110]. In breast cancer experimental models, inhibition of tumor growth and angiogenesis was observed after blocking the signaling function of TF but not its coagulation activity, and after inhibition of PAR-2, but not PAR-1 activity [109, 110]. A similar phenotype was observed in glioblastoma (GBM), which is the most aggressive primary brain tumor characterized by intense neovascularization, EC hyperplasia and hypercoagulation [73]. Experiments with GBM cell lines determined that there was expression of PAR-1 and PAR-2 in these cells as well as in vascular vessel walls within the invasive area of brain tumors [31, 72, 73]. However, only stimulation of the PAR-2 pathway led to increased secretion of VEGF and IL-8 suggesting that PAR-2/MAPK/ERK1/2, but not PAR-1/PI3K/Akt, signaling regulates angiogenesis in GBM. It is noteworthy that in GBM cells there is a correlation between TF and PAR-2 expression [72]. There is also evidence that hypoxia upregulates PAR-2 expression in brain tumors. There is an approximately 2.5-fold increase in PAR-2 expression in hypoxic vs. normoxic microvascular ECs of GBM, resulting in HB-EGF upregulation and a proangiogenic phenotype [111]. Poole *et al.* have recently demonstrated that PAR-2, which is a central factor in neurogenic inflammation and pain, sustains inflammation through a novel TRP channel-coupling mechanism. By generating bioactive lipids such as 5′,6′-EET and 12(S)-HETE, the proinflammatory effects of PAR-2 are sustained through TRPV4-dependent Ca^2+^ signals [112]. This may prove extremely relevant in this context as TRPV4 has been shown to impact angiogenesis at multiple levels [113, 114]. Finally, EGFR-induced signaling in glioma cells stimulates expression of TF, FVII, and PAR-2, thereby increasing TF/VIIa-mediated PAR-2 activation in cancer cells [115], and cancer cells may secrete aFVII that can act alone to activate PAR-2 [116].

TF/VIIa-mediated PAR-2 activation results in a transient increase in Ca^2+^ levels and triggers intracellular signaling that is dependent on the MAPK family (p44/42, p38, JNK), PI3, Src-like kinases, Jak/STAT, Rho GTPases, Rac1, and Cdc42 pathways [40, 80, 102]. In addition, elevated levels of proangiogenic proteins, such as VEGF, Cyr61, VEGF-C, CTGF, CXCL1, IL8, and immune modulators, such as GM-CSF (or CSF2) and M-CSF (or CSF1), have been observed [38, 68, 73, 97]. The efficacy of TF/VIIa/PAR-2-mediated activation of angiogenic mediators is greater than that induced by PAR-1 signaling [38]. TF-triggered PAR-2 signaling also results in increased MMP-9 expression, which positively correlates with the invasiveness of MCF-7 breast tumor cells [70] and may be linked to MMP-9 response to arachidonic acid metabolism [117]. It was reported that TF/FVIIa/PAR-2 interactions are critical for MDA-MB-231 breast cancer cell migration and invasion toward NIH-3T3 fibroblast-conditioned medium [68]. Therefore, TF/VIIa-induced PAR-2 activation facilitates proliferation and survival as well as metastatic potential of cancer cells [50, 68, 70].

In breast cancer cells, PAR-2 activation may also be induced by FXa as well as by TF/FXa or TF/FVIIa/FXa complexes. Subsequent MAPK phosphorylation or Erk1/2 activation then stimulates cancer cell migration and invasion [67, 68].

In tumors with high levels of TF (prothrombotic state), the predominant metastatic mechanism results from the coagulation activity of TF instead of its inherent signaling capacity [109]. The procoagulant activity of TF leads to thrombin generation, platelet activation, and platelet-dependent protection from natural killer cells as well as fibrin formation and monocyte/macrophage recruitment, all of which influence angiogenic and metastatic properties of the tumor [6, 97, 106, 118]. Studies of Yokota *et al.* [106] provided new insight into thrombin-mediated TF-dependent metastasis based on a hyperthrombotic mouse model with thrombomodulin deficiency (TM^Pro^ mice). TF-dependent, but contact-pathway-independent, breast cancer metastases were associated with hyperactivity of platelets and formation of platelet-leukocyte aggregates. Genetic deletion of platelet glycoprotein Ibα (GPIbα) and leukocyte CD11b excluded these receptors from platelet-dependent metastases. In addition, blockade of both host and tumor PAR-1 significantly decreased tumor cell metastatic potential. Similar results were obtained in melanoma models, thus confirming the contribution PAR-1 to melanoma and breast metastases [97].

### Thrombin

Generation of thrombin (IIa) is the central step in blood coagulation. As mentioned above, thrombin cleaves fibrinogen to yield fibrin and activates blood platelets resulting in the formation of an effective blood plug after vessel injury. However, enzymatically active thrombin is also detected in various types of surgically removed malignant tumors (e.g., small cell lung cancer, renal, ovarian, laryngeal, pancreatic, and gastric cancer, as well as melanoma) [11, 98, 119, 120].

The presence of TF on tumor cells contributes to thrombin generation in the tumor microenvironment independently of blood coagulation. Multiple thrombin targets (e.g., blood platelets and EC activation, fibrin generation) contribute to cancer progression by providing matrix for new vessels and metastatic tumor cell colonies [118, 121, 122]. The first reports of a novel role for thrombin in tumor cell metastases were published in the early 1990s [123–128]. When incubated with W256 carcinoma cells, α-thrombin produced a 50–300 % increase in adhesion to rat aortic endothelial cells and fibronectin [123–127]. Thrombin precursors and analogues including prothrombin, prothrombin-1, mesyl-thrombin, exo-site-thrombin, DFP-thrombin, and nitro-thrombin imitated the effect of α-thrombin [123–127]. Interestingly, α-thrombin coupled with its inhibitors, namely hirudin or antithrombin III-heparin complex, was not as effective at enhancing tumor cell adhesion as the native form of the enzyme [123–127]. The data indicate a new mechanism of thrombin interaction in tumor cell metastasis that is nonproteolytic. Moreover, mice transplanted with human ovarian cancer cells (SKOV3) demonstrated elevated tumor size and decreased survival rate when treated with thrombin [122]. Whether thrombin signaling works synergistically with the arachidonate metabolizing pathways that stimulate ovarian cancer growth remains to be determined [129]. In addition to its pivotal role in the coagulation pathway, thrombin is regarded as the main PAR-1 and PAR-4 activator. Thus, many cellular responses, including the ones observed in cancer cells such as cytoskeletal rearrangement [130], are thrombin-dependent. The evidence for a crucial role of TF-dependent thrombin generation and thrombin-mediated platelet PAR-4 activation in cancer progression and metastasis comes from studies performed on genetically modified mice. Stromal and tumor cells are involved in multiple steps of tumorigenesis, including proliferation, angiogenesis, invasion, and survival. Those animals depleted of platelets, PAR-4, or fibrinogen were protected from metastasis [118, 121, 122]. Treatment of melanoma B16a cells with α-thrombin resulted in a significantly increased number of metastatic lung colonies [123–127]. Prothrombin, ɣ-thrombin, and mouse thrombin, but not nitro-thrombin, were able to mimic the α-thrombin effect of enhancing lung colonization potential of tumor cells [123–127]. Administering thrombin intravenously with colon cancer cells (CT26) and melanoma cells (B16a) increased murine pulmonary metastases 4- to 413-fold [131]. The metastatic potential was diminished by hirudin, a specific inhibitor of thrombin [4, 122, 132].

### Thrombin/PAR-1 in fibroblasts

During the coagulation process the conversion of prothrombin to thrombin and its subsequent activity leads to cleavage of fibrinogen to form fibrin. Fibrin deposits in the tumor microenvironment are the store of thrombin that is released upon degradation of fibrin by plasmin [133]. The *in vitro* experiments provided evidence that stromal cells of malignant tumors, such as fibroblasts express elevated PAR-1 and PAR-2 compared to benign lesions or normal tissues where such expression is not observed [74]. Chronic PAR-1 mediated signaling in NIH-3T3 fibroblasts can cause growth transformation [85]. Reportedly, PAR-1 expression in the microenvironment drives progression and induces chemoresistance of pancreatic cancer [134] by regulating monocyte migration and fibroblast-dependent chemokine production.

### Thrombin/PAR-1 in endothelial cells

Endothelial cells are another target of thrombin/PARs interactions. Thrombin-mediated PAR-1 activation regulates inflammatory pathways that are also implicated in cancer progression. Increased lipid production and expression of PAF, IL-1, IL-6, IL-8, TNF-α and adhesive molecules (E-selectin, P-selectin, intracellular adhesion molecule-1 and vascular cell adhesion molecule-1, integrins) promotes EC proliferation, platelet recruitment, and malignant cell attachment [4, 5, 128, 135–138]. Inhibition of PAR-1 activity inhibits EC growth by increasing the sub-G0/G1 fraction, thereby reducing the percentage of cells in S-phase [139]. Moreover, thrombin/PAR-1 activation regulates barrier function between ECs by modulating adherens junctions (AJ) [140]. The increase in endothelial barrier permeability in response to thrombin/PAR-associated actions results from VE-cadherin, p120, and β-catenin modification via protein kinase C-dependent signaling [138, 140]. The dysfunction in the endothelial barrier generates a temporary proangiogenic matrix that is the basis for the activation of the thrombin/PAR/IP3/Ca^2+^/MAPK cascade and subsequent cellular responses [98]. Upregulation of angiogenic factors such as VEGF, VEGFR2, and angiopoietin-2 via the thrombin/PAR-dependent pathway together with enhanced barrier permeability of ECs results in the induction of angiogenesis and cancer dissemination [4, 141].

The integrin α_v_β_3_ is found mainly on blood vessel cells and plays an essential role in angiogenesis. Localization of α_v_β_3_ is altered in response to proinflammatory eicosanoid metabolites such as 12(S)-HETE leading to EC retraction and disruption in barrier function [142–144]. The expression of integrin α_v_β_3_ is regulated by thrombin-mediated PAR-1 activity. Thrombin activation of PARs also leads to increased expression of gelatinases that degrade collagen IV and increase vessel permeability to promote endothelial and cancer cell migration and invasion [120].

In ECs, thrombin can directly cleave PAR-1, which is thought to lead to a proinflammatory phenotype, or it can do so indirectly after it activates an intermediate protease called protein C (activated protein C (APC)) that then acts on PAR-1. However, when the GLA-domain of APC is in complex with its cognate receptor, EPCR, and thrombomodulin (TM), the signaling specificity of PAR-1 is altered to an anti-inflammatory or protective phenotype. Thus, in ECs, modulation of coagulation protease signaling specificity through PAR-1 depends on whether thrombin is acting directly on PAR-1, or indirectly, through APC, and whether APC is bound to EPCR [145]. In ECs, PAR-1 can be acted on by both thrombin and activated protein C (APC) to affect opposite outcomes, but this is thought to depend on whether the latter protease is in complex with EPCR.

The APC/EPCR/PAR-1 pathway induces motility, proliferation of ECs, and angiogenesis via vascular-protective signaling and tube formation to promote cancer cell dissemination [146, 147]. Moreover, the EC-associated modulator of hemostasis, TM, also strongly influences metastatic potential associated with thrombin procoagulant function [148].

Recent reports have linked PAR-2 and TRPV4 activation, where TRPV4 is known to enhance EC proliferation and arachidonic acid-mediated tumor EC migration [112, 113].

### Thrombin/PARs in platelets

Human platelets express two types of thrombin-triggered PARs, namely the high-affinity PAR-1 and low-affinity PAR-4. Both receptors activate pleiotropic cellular effects via coupling to protein Gα_q_ and Gα_13_, which leads to the activation of phospholipase Cβ, hydrolysis of phosphoinositides, and increased cytoplasmic calcium concentration, resulting in activation of integrin α_IIb_β_3_, and platelet aggregation [5, 6, 91, 149]. Initial reports describing the dual PARs system in human platelets explained this phenomenon by the fact that PAR-1 and PAR-4 interact with different concentrations of activator and thus may tune to thrombin signaling more efficiently [30]. Additional studies revealed that PAR-4 functions differently than PAR-1, in that thrombin-induced cleavage of PAR-4 results in much longer activation of Gα_q_. This leads to a sustained Ca^2+^ response, which prolongs secondary signaling, compared to PAR-1, which is crucial for the late phase of platelet aggregation [150]. At low thrombin concentrations, PAR-1 may act as a cofactor of PAR-4. There is also thrombin-mediated mitogenic PAR activity derived from platelets as well as for ECs and myocytes of vessels [97, 120]. Platelets coated with thrombin survive longer, which gives cancer cells opportunity to adhere and invade further [4, 120, 151]. Moreover, tumor cells coated by platelets are protected from natural killer cell-mediated elimination [152].

The aggregation of platelets and resultant fibrin generation is accompanied by increased expression of adhesive proteins (glycoprotein GPIIb/IIIa, von Willebrand factor, P-selectin, fibronectin) in platelets that have undergone thrombin stimulation [4]. These adhesive proteins enable malignant cells to form complexes with fibrin thrombus and blood platelets in vascular spaces in melanoma and epithelial cancers [reviewed in 4]. These complexes enhance cancer cell survival and metastatic potential. Thrombin treatment of platelets promoted melanoma cell adhesion to platelets, which increased lung metastasis [129].

In addition to platelet aggregation, thrombin-mediated PAR-1 and PAR-4 cleavage induces selective release of platelet proangiogenic and mitogenic regulators (PDGF, VEGF, and angiopoietin-1) that facilitate migration of endothelial progenitor cells and new capillary net formation, which is a pivotal step to metastases [153]. Compared to healthy subjects, platelets from breast cancer patients produce much higher levels of VEGF in response to thrombin stimulation [151]. Thrombin induced this effect through PAR-1 activation, while PAR-4 stimulation resulted in secretion of endostatin, an antiangiogenic factor [151].

### Thrombin/PAR-1 in cancer cells

Thrombin can elicit a signaling response via direct interaction with PAR-1 present on tumor cells [4, 14, 41, 48]. *In vitro* studies with various cancer cell lines showed correlation between overexpression of PAR-1 in cancer cells and greater invasiveness and development of distant metastases [14, 17, 18, 41–44, 52, 94, 154]. Moreover, in patients with lung, gastric, or breast cancer, PAR-1 expression was an independent, unfavorable prognostic factor in terms of overall survival, while in prostate cancer patients, it turned out to be a prognostic factor for local recurrence [17, 18, reviewed in 155]. Decreased expression of PAR-1 was associated with reduced invasiveness of cancer cells [68].

PAR-1 expression has been confirmed in melanoma, breast, lung, esophageal, gastric, colon, prostate, pancreatic, liver, ovarian, endometrial, and head and neck cancers (Table 1) [17, 38, 43–45, 78, 79, reviewed in 155–157]. Intriguingly, although PAR-1 is expressed in normal hematopoietic stem cells, its expression is markedly diminished in acute myeloid leukemia [158]. The cellular effect induced by PAR-1 depends on the concentration of agonist such that low concentration of thrombin (less than 3 nM) stimulates cancer cell proliferation and tumor growth, while high thrombin levels lead to apoptosis [159]. Most cellular effects are triggered via long-lasting activation of second messengers ERK1/2. However, multiple intracellular signaling pathways may be implicated in thrombin/PAR-1 activation (described below) [118, 160].

#### Apoptosis, proliferation, migration, and invasion

In murine models of benign tumors, PAR-1 activation results in tumor growth and invasion by silencing proapoptotic genes [154]. However, in epithelial cancers and melanoma cells thrombin-mediated PAR-1 activation triggers prosurvival pathways [5, 50, 75, 77, 154, 161]. Overexpression and activation of PAR-1 in nonmetastatic melanoma cell lines stimulates the Akt/PKB signaling pathway, leading to a decrease in Bim and Bax expression, as well as cleaved caspase-3 and caspase-9 levels. Inhibition of PAR-1 activity decreased tumor growth during *in vivo* experiments, confirming apoptosis-related effects elicited by this receptor [5].

In numerous cancers, the response to thrombin-induced PAR-1 activation increases cell proliferation, as well as motility and migration in Matrigel barrier assays [45, 46, 50, 77]. In Hep3B liver carcinoma cells, PAR-1 and PAR-4 activate common promigratory signaling pathways via activation of the receptor tyrosine kinases Met, PDGFR, and ROS kinase, as well as the inactivation of the protein tyrosine phosphatase, PTP1B [162]. In nasopharyngeal cancer, thrombin-induced PAR-1 activation leads to increased expression of MMP-2 and MMP-9, which are closely associated with tumor metastasis as they can degrade the extracellular matrix and disrupt the basement membrane [43, 60].

#### Increased expression of integrins

Integrins are transmembrane proteins that mediate the interactions between ECs and extracellular matrix that are vital for successful angiogenesis [41, 42, 120]. There is substantial evidence that enhanced expression of adhesion proteins due to thrombin-mediated PAR activity results in increased metastatic potential of cancer cells [4, 41–43]. PAR-1 increases the invasive properties of tumor cells primarily by promoting adhesion to extracellular matrix components. Several cancer cell lines (e.g., lung and melanoma) exhibit increased adhesion to platelets as well as aortic and capillary ECs after thrombin/PAR-1 stimulation [4, 14, 41, 42, 130]. PAR-1-driven adhesion to extracellular matrix components occurs via three mechanisms: (1) phosphorylation of focal adhesion kinase and paxillin, and induction of focal contact complexes, (2) mobilization of integrins on the cell surface without altering their level of expression, and (3) specific recruitment of integrin α_v_β_5_ to focal contact sites [163]. Interaction of cancer cells with integrin α_v_β_5_ and cytoskeletal reorganization facilitates cell migration, invasion, and metastatic development in lung cancer and melanoma [43, 163, 164]. Moreover, the application of anti-α_v_β_5_ antibodies specifically attenuates this PAR-1-induced invasion [163]. Expression of integrin α_IIb_β_3_ and P-selectin in response to PAR-1 may lead to attachment of melanoma cells to ECs and platelets and in this way also increase metastatic potential of cancer cells [14, 41, 42, 120]. Increased expression of α_IIb_β_3_ protein was reported in several malignant tumors [4, 14, 41, 42, 165, 166].

#### Angiogenesis

The development of new blood vessels, angiogenesis (*angio*—vessel, *genesis*—creation) is the pivotal process for tumor growth and progression [167, 168]. Small blood vessels provide cancer cells with oxygen and nutrients and remove metabolic waste products. It is assumed that malignant tumors cannot grow above 2–3 mm^3^ without vasculature [168]. Murine embryogenesis and cancer studies demonstrated that PAR-1 expression is necessary for angiogenesis as half the animal embryos deprived of PAR-1 died due to insufficient vasculature development, while activation of PAR-1 signaling prevented cancer cell death [169]. In melanoma and breast cancer cells PAR-1 expression correlates with increased VEGF levels, and stimulation of angiogenesis and tumor growth [161]. There is also correlation between thrombin and VEGF expression in glioma cells suggestive of a possible autocrine mechanism of regulation of angiogenesis in brain tumors.

Thrombin-mediated cleavage of PARs in cancer, blood cells, and vessel wall cells results in activation of transcription of many proangiogenic genes such as VEGF and its receptor (VEGFR), TF, MMP-2, angiopoetin-2 (Ang-2), basic fibroblast growth factor (bFGF), MAP, and PI3 kinases [120, 142, 170–173]. Based on *in vitro* studies, VEGF stemming from platelets and cancer cells may be secreted within minutes of activation [170]. Moreover, thrombin-mediated PAR activation induces production of reactive oxygen species (ROS) via increased expression of hypoxia induced factor-1 (HIF-1) [116]. HIF-1 activates VEGF gene transcription, and its expression is responsive to arachidonic acid metabolites [174].

PAR-1 and PAR-4 signaling after platelet activation leads to synthesis and release of thromboxane (TXA_2_) and 12-hydroxyeicosatetraenoic acid (12(S)-HETE) [6, 142, 143, 175–181]. These are metabolic end products of cyclooxygenase (COX-1) and lipoxygenase (12-LOX) activity on arachidonic acid and are important mediators of thrombus formation, vascular tone, and angiogenesis through their action on specific receptors (TPα, GPR31) and transcriptional regulation of factors such as VEGF and HIF1α [144, 174–186]. Arachidonic acid is released as a substrate for these enzymes from the cell membrane by cytosolic phospholipase A2 (cPLA2a) that responds to signaling from PAR-1 and PAR-4 differentially depending on whether it is coupled to the COX-1 pathway or the 12-LOX pathway [187]. Thrombin activation of PAR-1 and PAR-4 also leads to the formation of esterified eicosanoids at the same rate as the release of free acids. However, HETE esterified to phosphatidylethanolamine after this reaction gets presented to the cell exterior instead of recycling in the interior substrate pool and has unique functions in that context [188].

#### Epithelial–mesenchymal transition

Another potentially important phenomenon in cancer metastasis, at least in part regulated by thrombin, is epithelial–mesenchymal transition (EMT) [44]. The mechanism, with its reverse process, a mesenchymal–epithelial transition (MET), enhances the ability of solid cancers to disseminate and colonize distant sites [189]. Malignant tumors composed of moderately differentiated cells can also contain regions of poor differentiation. These cells may detach from the tumor mass and invade the adjacent stroma after undergoing an EMT-like event. They lose expression of epithelial differentiation markers and gain the capacity to express mesenchymal and “stemness” markers. These cells also contribute to migrating circulating stem cells (CSCs) that disseminate and give rise to metastases. During EMT, some characteristics of differentiated epithelium (e.g., apico-basal polarity and cell–cell adhesions) are replaced with mesenchymal traits—rear to-front polarity, capacity for individual cell migration, and invasion of basal lamina and blood vessels [189]. To effectively colonize new sites, such cells must also be capable of undergoing the reverse MET process to re-differentiate and re-establish the organization of cells [189].

Experimental studies on gastric cancer cell lines revealed that thrombin-mediated PAR-1 activation leads to reprogramming of gene expression by stimulation of transcription factors like SNAIL1 that is known to drive EMT in the embryo [44]. Moreover, in epithelial cancers (e.g., gastric and breast), the thrombin/PAR-1 complex leads to alteration in basement membrane components (increased expression of fibronectin, Wnt and β-catenin, decreased expression of E-cadherin) as well as cytoskeletal proteins (myosin IIA and filamin B), which collectively regulate EMT involved in malignant tumor progression [45, 46, 75, 77, 94, 189].

### MMPs

MMPs are zinc-dependent proteases secreted by both tumor and host cells. It is widely recognized that MMPs are involved in cancer progression and metastasis by facilitating tumor cell invasion through the basement membrane and stromal tissue [39, 157, 190]. Coexpression of MMPs and PARs is associated with high invasiveness (deeper infiltration of tumor, lymphovascular invasion, more frequent occurrence of lymph node metastases, more advanced clinical stage of the disease) and poor survival in several malignant tumors, e.g., breast, gastric, esophageal, gallbladder, hepatocellular, lung, and ovarian cancers [18, reviewed in 39, 157, 189].

In addition, studies with breast, gallbladder and ovarian cancer cell lines have shown that MMPs (MMP-1, MMP-9, MMP-13, MMP-14) may activate PARs signaling, especially by cleavage of PAR-1 (majority of tumors) or PAR-2 (lung cancer) [15, 39, 52, 71]. Moreover, it was determined that senescent fibroblasts enhance early skin carcinogenic events via MMP-1-mediated PAR-1 activation [191]. Of the MMPs tested, MMP-1 presents the strongest positive correlation with cell migration and invasiveness. The blockade of MMP-1-mediated PAR-1 activity in xenograft models of advanced peritoneal ovarian cancer results in the inhibition of angiogenesis and metastasis [39].

Activation of platelet PAR-1 by MMP-1 can also lead to Rho-GTP as well as MAPK signal activation, thereby promoting platelet aggregation as well as increasing platelet motility and cell proliferation [87]. The ProMMP-1 zymogen is converted to MMP-1 on the platelet surface after contact with collagen fibrils. Blockade of MMP-1/PAR-1 signaling greatly inhibits thrombosis in animals, demonstrating that the collagen/MMP-1/PAR-1 pathway is an activator of platelet signaling events independent of thrombin. As PARs stimulate the expression and release of 12(S)-HETE that upregulates MMP9 [117], there appears to be a precedent for bi-directional regulation of MMPs and PARs signaling.

### Trypsin

Trypsin is another serine protease that activates PAR-2 in cancer cells. The concentration of trypsin is increased in patients with gastric, colon, pancreatic, and ovarian cancer [54, 155, 192]. Increased expression of PAR-2 and its influence on cancer cell proliferation was defined in gastric, esophageal, colorectal, pancreatic, oral squamous, liver, cholangiocarcinoma, lung, breast, and ovarian cancers, as well as in melanoma and brain tumors (Table 3) [53, 54, 56, 57, 60, 61, 64, 65, 71, 155, 193–195]. PAR-2 may be highly expressed in stroma-rich tumor regions also. Studies by Shi et al. have demonstrated intriguing dual roles for stromal PAR-2 in pancreatic cancer development, namely that PAR-2 potentiated primary tumor growth but diminished lymphangiogenesis and subsequent lymph node metastasis [194]. The findings defined PAR-2 as a negative regulator of lymphangiogenesis in pancreatic cancer. In contrast, the expression of PAR-2 correlated with the depth of wall invasion, liver metastasis, as well as lymphatic and venous infiltration in gastric cancer patients [193]. Patients with PAR-2-positive tumors had significantly poorer prognosis than those with expression-negative tumors.

*In vitro* studies with epithelial cancers have shown that PAR-2, like PAR-1, exerts mitogenic activity [46, 53, 54, 56–61, 64, 71, 155, 193–195]. Trypsin and PAR-2 activating peptide, SLIGKV, significantly increased gelatinolytic activity of MMP-2, as well as ERK/AP-1, MEK1/2, and MAPK signaling to promote cancer cell proliferation, migration, and metastasis [53, 57, 58, 60–62, 195]. The increased activity of MMP-2 suggests that PAR-2 may be implicated in cancer invasion by the MMP/EGFR/MAPK/ERK1/2 pathway [60]. PAR-2 may also activate Ca^2+^ channels to promote prostaglandin E2 release resulting in EGFR-stimulated cell proliferation [53]. Employing a migration assay through Matrigel barrier, it was determined that the Met receptor tyrosine kinase transactivation by PAR-2 is involved in hepatocellular and cholangiocarcinoma cell invasion [58].

The influence of inflammation in cancer is undeniable. There are interesting connections between the nervous system and regulation of inflammation, where the vagus nerve participates in a systemic feedback loop that also involves PARs [reviewed in 196]. Recent studies determined that the PAR-1 isoform on vagal C-fibers in mouse lungs could evoke an action potential in response to thrombin, trypsin, or the PAR-1-activating peptide TFLLR-NH(2) [197]. The TRPV channels that induce pain and inflammation are also regulated by the PARs and their downstream proinflammatory bioactive lipid mediators such as 12(S)-HETE [198–204]. While we mostly associate neurogenic inflammation with nociception, it should be noted that tumor cells can migrate via a perineural route, which may speak to the proinflammatory PARs-bioactive lipid gradients along the nerves serving as metastasis beacons [205–207]. Similarly, neurogenic mechanisms have been described that relate PARs activation to extravasation of plasma and that depend on bioactive lipid mediators [112, 208]. Biopsies around the Bartholin gland of women with vestibulodynia reveal more intraepithelial nerve endings than healthy individuals and increased release of inflammatory mediators that lead to C-nerve fiber sensitization and increased proliferation [209]. Because of this neurogenic inflammation, these patients typically experience recalcitrant yeast infections that can lead to epithelial hyperplasia and cancer [210].

### Microbiome, PARs, cancer

PARs have been implicated in many host–microbe interactions that in time may prove relevant to deciphering the role of microbiome in cancer onset and progression as well as other diseases with roots in infectious inflammatory processes [211–217]. Microbial insult by *Streptococcus pneumoniae* is known to stimulate host-derived proteases so as to activate PARs [218]. *Porphyromonas gingivalis* can activate PARs on oral epithelial cells to upregulate IL-6 [219], and the bacterium was recently demonstrated to stimulate PAR-2 resulting in MMP9 expression and promotion of oral squamous cell carcinoma [220]. Both *Streptococcus pyogenes* and *Staphylococcus aureus* on the skin produce proteases that fuel the activation of PARs on keratinocytes leading to inflammation [221]. Microbes themselves produce numerous proteases that aid in microbial dissemination by overcoming some of the same logistical processes that metastasizing cancer cells must circumvent to spread [222–225]. The interplay between microbiome and host to affect changes in tissue and hematologic microenvironment are actively being investigated [226, 227]. Bacterial proteases can cleave PARs to modulate inflammation and have been studied for their potential to compromise host barrier function [228]. To that end, it is conceivable that circulating or metastasizing cells from tumors or stem niches could take advantage of such changes. Recently, there is also evidence for microbial protease activation of a novel TLR in a mechanism similar to PAR activation [229].

As food for thought, the gut microbiome has received a lot of attention in relation to disease and well-being [230–233]. Therefore, it is noteworthy in the climate of genetically modified foods that are either bred or engineered that bountiful yields of certain grains in the crop industry rely on serpin expression [233, 234], which may have implications for PAR regulation in the gut [235–239].

### Clinical implication

Results of theoretical studies presented above suggest that PARs and PARs-associated signaling may be used as a possible therapeutic target, either alone or in combination with other modalities, such as chemotherapy, antiangiogenic agents, and proapoptotic drugs. A PAR-directed approach is appealing since it targets both the tumor and its microenvironment. *In vitro* and *in vivo* studies provide evidence that inhibition of PAR-associated signaling results in reduced tumor growth, invasiveness, and metastasis [5, 41, 42, 240]. There are functional (inhibitors of proteases) and pharmacological (inhibitors of tethered ligand or cleavage site of PAR) PAR-associated signaling antagonists [19, 20]. Clinical benefit may be provided by direct blockade of PAR-1 or PAR-2 on tumor cells, inhibition of PAR-1 on platelets, fibroblasts, and ECs (ATAP2, WEDE15, SCH530348, SCH79797, vorapaxar), as well as administering inhibitors of thrombin (hirudin, argatroban), TF (TFPI, mAb-10H10), MMPs, and other serine protease inhbitors (serpins) [4, 5, 16, 22, 24, 87, 95, 96, 241, 242]. Although experimental trypsin inhibition is feasible, it seems that trypsin as a target for clinical therapy is unlikely to be successful due to its universal distribution [60]. The blockade of proteins expressed in response to PAR-elicited signaling, e.g., anti-α_v_β_5_ antibodies, EGFR, Erb, Erk, MEK inhibitors, as well as agents interfering with PAR RNA (short hairpin RNA (shRNA)), also have therapeutic potential [24, 61].

The inhibition of related activities that are not associated directly with cancer-promoting effects of PARs may also benefit cancer patients. There are intriguing findings from an animal model that thrombin-mediated PAR-1 and PAR-2 activation plays a role in the pathogenesis of acute side effects of radiotherapy, e.g., enteritis, where PAR-mediated signaling activates inflammatory, mitogenic, and proliferative processes in cells of the gut after radiotherapy. PAR-1 inhibitors decreased intensity of acute, immediate-early side effects (enteritis), but did not affect late-onset side effects [243–245]. The pathogenesis of late adverse effects is presumed to be PAR-independent. Moreover, PAR-2 antagonists potentiate analgesic effects of systemic morphine in a rat model of bone cancer pain [246].

Although results from experimental models are promising, inhibition of PAR activity on both normal and tumor cells may cause side effects, such as hemorrhage, so that PAR-tailored drug discovery is a great challenge. Clinical trials are still limited and so far directed to patients with diseases other than cancer. PAR-1 antagonists, such as vorapaxar and atopaxar, have been assessed in clinical trials in patients with acute coronary syndrome, cerebral infarction, and atherosclerosis [24, 247].

However, insight into the molecular basis of breast cancer and melanoma provides new potential targets for anticancer drug discovery tailored to PAR-dependent signaling.

### Breast cancer

There is growing evidence that PARs, mainly PAR-1 and PAR-2, are strong mediators of cell invasion in epithelial cancers [68, 77]. Breast cancer cells may express both PAR-1 and PAR-2 [66, 68, 77], and their role in breast carcinoma is the most widely studied. PAR-1 is not expressed in normal breast epithelium, dysplasia, or adenoma but is upregulated in carcinoma *in situ* (low expression) and is highly expressed in invasive breast carcinoma cell lines [47, 77, 154]. Experimental studies on breast cancer have shown that PAR-1 is activated by thrombin, MMPs and TF, while PAR-2 is activated by coagulation factors VIIa, Xa, or their complexes with TF [16, 52, 66, 68, 77]. There are also observations that PAR-1 and PAR-2 act as a functional unit in this tumor type [248]. Silencing PAR-2 by shRNA attenuates thrombin-mediated PAR-1 activation, leading to reduced colony formation and decreased cell invasion [248].

PAR activity mediates breast cancer cell migration through Matrigel (a reconstituted basement membrane), facilitates cell chemokinesis through the Gαi/c-Src/JNK/paxillin signaling pathway, activates Akt-dependent survival pathways, and correlates with the level of invasiveness and metastatic potential of numerous cancer cell lines [66, 77, 154, 163]. PARs also regulate EMT processes in breast cancer tumors, which facilitates cell proliferation (*in situ* carcinoma), encroachment of basement membrane, matrix degradation, and local infiltration (invasive cancer). Furthermore, PAR interactions with integrins, formation of focal contact complexes, and cytoskeleton reorganization enable distant dissemination via intravasation and extravasation (via lymphatic or blood vessels). Finally, the MET process, and interactions with blood and ECs, facilitates metastases formation (disseminated cancer) [77]. Inhibition of PAR activation in highly metastatic MDA-435 breast cancer cells reduced cell invasion [77]. Administering an MMP-1 inhibitor and P1pal-7 (inhibitor of cell viability mediated by Akt signaling) attenuates Akt activity, significantly promoting apoptosis in breast tumor xenografts and inhibiting metastasis to the lungs by up to 88 % [16].

There is evidence from *in vivo* studies for PAR-mediated breast cancer progression [32]. PAR-1 expression was essential for tumor growth and invasion in mammary xenografts via thrombin-mediated interaction with EGFR- and ErbB or by the fibroblast-derived MMP-1-mediated Ca^2+^ pathway [32, 52]. Persistent transactivation of EGFR and ErbB2/Her2 by the thrombin-cleaved PAR-1 pathway has been demonstrated in invasive breast carcinoma, but not in normal mammary epithelial cells [32, 94]. There is evidence that Gαi/o, metalloprotease activity and release of HB-EGF (*heparin-binding EGF*) ligand are critical for transactivation of EGFR. Finally, EGFR and ErbB2/Her2 signaling triggered by PARs results in prolonged Erk-1/2 activation leading to breast carcinoma cell invasion. These results indicate potential therapeutic benefit of inhibitors of thrombin, EGFR, ErbB and Erk kinases in metastatic breast cancer patients.

### Melanoma

In epithelial cancers, the predominant mechanism leading to metastatic dissemination is EMT. In melanoma, the transition of a lesion from the noninvasive radial growth phase (RGP) to the invasive and metastasis-competent vertical growth phase (VGP) is a major step in tumor progression, and PAR expression is implicated in the RGP-VGP transition process [249]. Melanoma cells express both PAR-1 and PAR-2 [50, 250]. The PAR-2 role in melanoma metastasis was not previously appreciated, but the newest findings have shown its dual role in melanoma [187, 194]. In a murine model of spontaneous metastatic B16 melanoma, PAR-2 contributed to the limitation of local cancer progression in one area, while enhancing distant metastatic spread. Numerous reports document the role of PAR-1 signaling in the prometastatic phenotype of melanoma cells [4, 5, 21]. Experimental studies on melanoma cell lines demonstrated that PAR-1-elicited signaling activates adhesive, invasive, antiapoptotic, and angiogenic factors to promote melanoma metastasis [4, 5, 21, 251]. Additional proof for the role of PAR-1 in melanoma dissemination is the fact that it is highly expressed both in metastatic melanoma cell lines and in metastatic lesions in comparison to primary nevi and normal skin [21, 250]. Moreover, melanoma cells isolated from lesions giving rise to metastases in patients had higher PAR-1 mRNA and protein expression, as compared to those obtained from lesions that did not develop metastatic disease [252]. Motility and migration of melanoma cells is also regulated by thrombin-mediated PAR-1 activation [50, 252]. Thrombin, whose generation is TF-dependent (procoagulant expressed in melanoma cells), is the predominant PAR-1 activator [21, 107]. However, there is also evidence that MMP-1-mediated PARs activation exists in melanoma cells [5, 249]. Both MMP-1 and PAR-1 are highly expressed by VGP melanomas. MMP-1 is thought to facilitate melanoma invasion by degrading type I collagen within the skin, while PAR-1 activation leads to increased activation of growth factors: FGFR-2 and IGF-1 [5, 249].

Experiments with the B16F10 murine metastasis model of melanoma demonstrated that cells transfected with PAR-1 exhibited substantially higher pulmonary metastasis potential than those deprived of PAR-1 signaling [4, 48]. PAR-1 promoted metastatic melanoma by regulating the tumor suppressor Maspin and the gap junction protein Connexin 43. Villares *et al.* [253] determined that Connexin 43 facilitates interaction between malignant cells and ECs, and maspin expression is decreased in metastatic melanoma cells, where there is an inverse correlation between PAR-1 and Maspin expression [254]. PAR-1 also promotes expression of melanoma cell adhesion molecule MCAM/MUC18 (MUC18), which is a key marker of melanoma metastasis. It is of interest that PAR-1 activity increases expression of platelet-activating factor receptor (PAFR) and its ligand, and so not only promotes platelet aggregation but also enhances MUC18 levels. This is extremely relevant to the metastatic process as it was demonstrated that the PAR1/PAFR/MUC18 pathway mediates melanoma cell adhesion to microvascular ECs, transendothelial migration and metastatic retention in the lungs [251].

PAR-1 silencing and thrombin inhibition affects the ability of metastatic melanoma cell lines to disseminate [21, 22, 251]. Inhibition of PAR activity by 80 % through the use of lentiviral shRNA decreases lung metastatic potential of PAR-1 overexpressing melanoma cell lines [21]. PAR-1 silencing also inhibits expression of the adhesive protein MUC18, which attenuates the metastatic phenotype of melanoma cells [251].

To reduce the toxic immune responses of viral therapy, PAR-1 small interfering RNA (siRNA) incorporated into neutral liposomes (1,2-dioleoyl-sn-glycero-3-phosphatidylcholine, DOPC) was used in experiments on melanoma models. There was a significant decline in tumor growth, weight, and formation of metastatic lung colonies in mice treated with the PAR-1 siRNA-DOPC [21]. siRNA delivery also resulted in a decline in VEGF, IL-8, and MMP-2 expression levels, and decreased blood vessel density. In another study, the reduction of PAR-1 expression by siRNA and the inhibition of PAR-1 function by the specific antagonist SCH79797 significantly decreased melanoma cell motility and invasiveness to the extent of the non-metastatic and low PAR-1 expressing cells [252]. A specific thrombin inhibitor, argatroban, also decreases migration and bone metastatic potential of B16BL6 melanoma cells [22].

These findings suggest that PAR-1-dependent stimulation of tumor growth and metastasis is regulated by invasive, adhesive and proangiogenic factors and that PAR-1 could be a potential therapeutic target for metastatic melanoma patients.

### Summary

Tumor cell invasion and metastasis involves complex interactions between mesenchymal cells and extracellular matrix as well as blood components and ECs. The coagulation proteases, matrix metalloproteases and serine proteases interact with PARs, thus promoting multiple activities leading to cancer progression. Further studies are necessary to convert theoretical knowledge into practical value.
